# The enigmatic links between Epstein-Barr virus infection and multiple sclerosis

**DOI:** 10.1172/JCI160468

**Published:** 2022-05-02

**Authors:** Kenneth L. Tyler

**Affiliations:** Departments of Neurology, Medicine and Immunology-Microbiology, and Neuroinfectious Diseases Group, University of Colorado School of Medicine, Aurora, Colorado, USA.

Speculation about a potential link between multiple sclerosis (MS) and infectious agents has existed since the mid-19th century. In 1877, Jean-Martin Charcot succinctly commented, “What is known in reference to the conditions that preside over the development of disseminated sclerosis comes to very little” (1). He noted that “an etiologic cause which deserves mention” is the influence of certain acute diseases and then cited reports of illness during convalescence from typhoid fever, on recovering from an attack of cholera, soon after a severe attack of smallpox, and after experiencing an unnamed febrile disease accompanied by diarrhea. In the modern era, studies linking MS to prior infection have generally focused on viruses, in particular the human herpesvirus family, and most recently on Epstein-Barr virus/human herpesvirus 4 (EBV).

## Mounting evidence connecting EBV to MS pathogenesis

A recent epidemiological study has generated intense new interest in the EBV/MS linkage (2). Bjornevik and colleagues took advantage of stored serum samples from over 10 million racially diverse, active-duty military personnel that had been collected over two decades (1993–2013) and stored in the Department of Defense Serum Repository (DDSR). Of 955 individuals that subsequently developed MS, 801 had serum samples available to assess their pre-MS EBV infection status, and 800 of these 801 sera, collected a median of 1 year before MS onset, were EBV seropositive. Overall, 95% of first-collection serum specimens in the DDSR were EBV seropositive, reflecting the ubiquity of EBV infection; however, the HR for MS incidence comparing those who were EBV^+^ versus EBV^–^ on initial serostatus was 26.5-fold higher. Thirty-four of the 35 (97%) initially EBV-seronegative subjects who went on to develop MS seroconverted before onset of their MS. CMV seroconversion, used as a control, occurred at a similar rate in those who developed MS and those who did not. Subsequent risk of developing MS was actually higher among those who were CMV seronegative compared with those who were seropositive.

A linkage between EBV seropositivity and MS has been repeatedly found in other studies. One meta-analysis of published papers on EBV serology in adults with MS encompassing 6700 subjects found that 93% were EBV seropositive compared with 86% of age-matched controls. The data in children were even more striking. In 759 children with MS, 85% were EBV seropositive compared with only 51% of age-matched controls (3).

Tightening the link of causality between EBV infection and MS requires that infection be an antecedent to MS, not just an accompaniment. In the DDSR study, the authors used sequential serum specimens to conclude that MS onset typically occurred a median of 5 years after the first documented EBV^+^ sample and approximately 7.5 years after seroconversion (2). EBV seroconversion also preceded elevations in serum neurofilament light chain (sNfL), an early biomarker of neuroaxonal injury in MS (4). This finding is consistent with prior work indicating that, among patients with the earliest diagnosable forms of MS, all of 901 subjects were EBV seropositive (5). These and other studies suggest that MS rarely, if ever, develops in the absence of EBV infection and that EBV infection is a necessary prerequisite for disease. The ubiquitous nature of EBV infection also indicates that, although EBV infection may be necessary to cause MS, infection alone is insufficient, as the overwhelming majority of EBV-infected individuals never develop MS. In this regard, some studies have suggested that specific characteristics of primary EBV infection, such as the presence of symptomatic infectious mononucleosis (6) or the nature of the host anti-EBV antibody response, notably to EBNA-1, are associated with heightened subsequent MS development risk (7). These and other EBV infection features, including EBV DNA copy number and expression of specific EBV microRNAs, likely act additively with other known MS risk factors, including HLA genotype, in determining MS risk (3, 8, 9).

## The missing mechanistic link

The next step in understanding the EBV/MS association is to identify biologically plausible mechanisms by which EBV infection could contribute to MS pathogenesis. This step is critical for rational design of EBV-focused disease-modifying therapies. The most frequently suggested candidate mechanisms are (7, 10) (a) CNS invasion with direct or indirect viral injury to oligodendrocytes, (b) upregulation of EBV-activated autoreactive T and B cells that migrate to the CNS and cause injury, and (c) molecular mimicry in which EBV infection induces antibody or T cell responses that are directed against viral antigens or epitopes but that crossreact with myelin or other CNS antigens.

In terms of the direct viral invasion hypothesis, attempts to detect EBV genome or antigen in brain tissue of MS patients as compared with controls have yielded inconsistent results. One study detected EBV by in situ hybridization in 82% of postmortem MS brain tissues as compared with 24% of controls and by PCR in 64% of MS brains compared with 24% of controls (11). However, other studies using apparently similar techniques have not found this association (see ref. 12). When EBV DNA or antigen is reported to be present in the CNS, it is typically found in infiltrating inflammatory cells, including B cells and plasma cells as well as EBV-specific CD8^+^ T cells, rather than in neurons, oligodendrocytes, astrocytes, or microglia (13, 14). When EBV gene expression is analyzed in MS brains in laser-capture microdissected areas of prominent immune cell infiltration, the upregulated genes detected are more commonly those involved in EBV latency rather than lytic infection (14). Even when EBV-infected astrocytes or microglial are reported, they clearly are a rare population (<15% of infected cells; ref. 11). An intriguing, though perhaps not absolutely en pointe observation, is the report of a spontaneously occurring MS-like demyelinating encephalomyelitis in Japanese macaques linked to Japanese macaque rhadinovirus, a primate gamma2-herpesvirus phylogenetically related to EBV, in which virus can be cultured from acute demyelinating lesions (15). These studies suggest that a direct invasion model should perhaps not be absolutely dismissed despite conflicting and ambiguous supportive evidence.

Molecular mimicry occurs when peptides encoded by a pathogen such as a virus share sequence or structural homology with host self-proteins. An immune response against the pathogen protein triggers a crossreactive response against the self-antigen, leading to tissue injury. The possibility that such a mechanism could occur in MS was first posited by Fujinami and Oldstone, who identified an amino acid homology between part of the hepatitis B virus polymerase and an encephalitogenic site of myelin-basic protein (16). It was subsequently noted that myelin-basic protein–specific T cell clones derived from MS patients could be activated by specific viral peptides, including one from EBV (17). Conversely, EBNA-1–specific CD4^+^ T cells isolated from MS patients can recognize myelin antigens and produce proinflammatory cytokines, including IL-2 (18), adding plausibility to the EBV/MS molecular mimicry theory. Most of the attention has been focused on the EBV EBNA1 protein; however, other EBV proteins, including BFRF3, have also been implicated in molecular mimicry (19).

EBNA1 has a region of high-sequence similarity with anoctamin 2 (AN02), and antibodies to either homologous region are crossreactive (20). AN02 is a Ca2^+^-activated chloride channel transmembrane protein expressed in neurons and glial cells. The homologous region of EBNA-1 is within an area against which higher antibody titers have been linked to increased risk of MS (9). Patients with MS have higher antibody levels to AN02 compared with controls, and these autoantibodies can be detected in cerebrospinal fluid (CSF) (20).

The stress-regulated heat shock protein αβ-crystallin (HSPB5) is another candidate molecular mimicry protein. This protein is upregulated in lesional and nonlesional white matter areas in brains of those with MS and is expressed in oligodendrocytes, astrocytes, and some demyelinated axons (21). This protein has protective and therapeutic roles in mouse experimental autoimmune encephalomyelitis (EAE) models, and mice null for the protein show more severe clinical and pathological EAE (22). A study screening serum and CSF antibody reactivity from MS patients that differs from those of controls using high-density peptide microarrays with protein sequences from candidate MS autoantigens found the highest signal was directed against an EBNA1 region that is homologous to the N-terminal region of αβ-crystallin (23). This protein is also expressed in oligodendrocytes, and it has been postulated, although proof is lacking, that the immune response against this protein could lead to oligodendrocyte injury and demyelination (7, 10).

The newest candidate for MS molecular mimicry is glial cell adhesion molecule (GlialCAM; ref. 24). This protein is an immunoglobulin superfamily member expressed in both astrocytes and oligodendrocytes and can be detected in active MS lesions. Monoclonal antibodies created from CSF of 6 of 9 MS patients’ sequenced B cell receptor (BCR) heavy and light chain VDJ region sequences bound to EBNA1. One created monoclonal antibody was used on several high-throughput proteomic platforms, including proteome array phage displays, to probe large human proteome arrays and was found to also bind to the C-terminal portion of the intracellular domain of GlialCAM, which overlaps a region that closely resembles an EBNA1 domain previously linked to MS risk (9). It is important to note that crossreacting EBNA1-GlialCAM antibodies were found in only about 25% of MS patients. An important aspect of this study was that the investigators tried to establish pathogenetic plausibility of this crossreactivity in an EAE model. They first immunized mice with an EBNA1 fragment, then induced EAE with a second immunization using a proteolipid protein (PLP) fragment. Mice developed robust antibody responses against the immunizing PLP fragment and the cognate GlialCAM intracellular domain. The EBNA1-immunized mice had more severe clinical (paresis) and neuropathological (immune cell infiltration and demyelination) disease than controls immunized with scrambled control EBNA1 peptides.

Taken together, these studies suggest that EBV infection may induce immune responses, particularly but perhaps not exclusively against EBNA1, that generate potentially self-reactive autoimmunity that could contribute to MS pathogenesis. However, even for the most promising candidate targets, including GlialCAM and AN02, antibody responses are only detected in 15% to 25% of MS patients (20, 24), suggesting that there is not a universal or even prevalent mimicry mechanism likely to be operative. It is critical to recognize that the demonstration of molecular mimicry is not in and of itself proof that this mimicry plays any role in the pathogenesis of MS.

## Therapeutic implications

Given the fact that EBV infection is both ubiquitous and often asymptomatic and the lack of any currently licensed EBV-specific antiviral drugs, attacking acute infection would not seem particularly promising as a therapeutic strategy. Similarly, lessons from other herpesviruses would suggest antiviral therapy is unlikely to eliminate latent virus, although they may suppress reactivation from latency. Perhaps not surprisingly, small uncontrolled trials performed to date of antiviral therapies in MS have suggested limited potential efficacy, but the drugs tested often have had suboptimal efficacy against EBV (25). Interestingly, anti-CD20 therapies are effective and have been licensed for reducing EBV viral loads in blood in EBV-associated hemophagocytic lymphohistiocytosis and posttransport lymphoproliferative syndromes, and it has been speculated that a similar anti-EBV therapeutic effect could be operative in MS (26).

Preventing an EBV infection entirely through vaccination is likely a more promising strategy than antiviral chemotherapy and offers the advantage of also targeting EBV-associated malignancies and potentially affecting other autoimmune diseases with which EBV has also been associated. The first trial of a human mRNA (mRNA-1189) vaccine developed by Moderna that encodes five EBV envelope proteins (gp42, gp350, gB, gH,and gL) to prevent infectious mononucleosis (ClinicalTrials.gov NCT05164094) just began enrollment in January 2022. Natural infection with EBV induces both humoral and cell-mediated immune responses, and adoptive transfer of EBV-specific T cells has been tested in treatment of nasopharyngeal carcinoma and lymphoproliferative diseases (27). Small scale uncontrolled human trials in MS have not resulted in serious adverse effects and have shown sustained benefits for up to three years in individual patients after therapy (28, 29). Future work examining whether mRNA or other vaccines that generate broad and effective humoral and cell-mediated immunity against EBV reduce risk of MS will be technically challenging and require long-term follow-up, but will be key to solving the longstanding mysteries surrounding MS and EBV infection.

EBV is clearly linked to MS, and accumulating data suggest it is both a necessary and early factor in the initiation of disease. Plausible biological mechanisms for EBV’s pathogenic role in MS have now been elucidated, but a universal unifying mechanism has not yet been identified. Therapeutic tools, including a new multivalent mRNA vaccine, are undergoing human trials in preventing EBV infection and disease, and if found to be safe and effective, this would open the door for groundbreaking clinical trials on the prevention of MS.

## Version 1 05/02/2022

Electronic publication
